# Transactions of the Brooklyn Dental Association

**Published:** 1864-09

**Authors:** W. C. Horne


### TRANSACTIONS OF THE BROOKLYN DENTAL ASSOCIATION.

REPORTED BY DR. W. C. HORNE.

June 10th, 1864.

Pursuant to an invitation from Dr. J. H. Smith, the Society represented by fifteen of its members, took the New Haven steamboat from New York at three o'clock P. M.; and after a delightful voyage of five hours, diversified by professional chat and non-professional joking, arrived at the New Haven wharf, where they were awaited by Dr. Smith and piloted by him to his residence. Here were assembled the members of the New Haven Dental Society, by whom we were cordially received. The meeting having been called to order by the President, Dr. Parks, and the usual preliminary business gone through with, the report of the Committee on Dental School was called for.

Dr. Atkinson explained, for the information of the New Haven friends present, that the subject of founding a Dental School in New York, had been for some time agitated among us; the workers, men desirous of progress, were the ones who had taken hold of the matter. They felt that it was of the first importance to have an Institute on which to rely for the proper education of students and young practitioners.

Ours was a young profession; he easily recalled the time when to be a Dentist was next thing to being a blackguard; now see the position that has been attained; there was no heart but would leap with joy at the achievement. In a good cause one man was better than a host of opponents; he would rather be right and do battle alone, than be pampered and fawned upon, without this great and unfailing support. But now we have such strength and skill that, standing together as a band of brothers, our ranks can not be broken. The American Dentists had never sent their best men abroad; what might we not expect if we should send out some of our number. Let us plant an acorn to-night which shall grow and flourish, through future generations.

Dr. C. P. Fitch reported a resolution which had been adopted by the New York Dental Society, to the effect that the New York and Brooklyn Societies desired the establishment of a Dental School during the winter of 1864-5. He said that the adoption of this resolution grew out of the conviction that a great need existed in the city of New York for a Dental School. In that city and its dependencies, Brooklyn and Jersey City, there were many Dentists and Dental students; these need a school at home; and the only wonder was that this want, so long felt, had not been yet supplied. The reason of this lay doubtless in the fact that heretofore, to a great extent, every one had been for himself. The effect of the contemplated institution would be to increase fraternal feeling, to erect a high standard of professional honor, and to elevate both the Dentists and the community.

Dr. Marvin was very glad to favor the course indicated, Dentists for the past have been too prone to stand alone, they have had too little association. There as been a disposition on the part of older members to shut in their light within their own circle. To-day our efforts have a single object, the advancement of the profession; and this we will pursue without fear of being frowned on or ridiculed. It is time for us to stand up and convince these gentlemen that they are not the whole profession. We are willing they shall receive of our light; we do not claim to possess everything. Our purpose is to set our standard so high that we may always be able to look up to it.

The resolution was then adopted and the Committee ordered to further consider the subject and report from time to time.

Dr. Atkinson then read a paper on "Dental Charges."

Dr. C. P. Fitch heartily concurred in the sentiments of the paper; the Dentist's charges turn on the estimate he makes of his services. When the patient comes to learn that teeth can be saved only by the best ability, he will demand the best service that can be had. When Dentists are willing to have their work submitted to their fellows, it is a pretty good sign that their work is about right. The demand the world has on us is more than personal feeling; we must be willing to learn; and when the community come to find what can be done, they will demand nothing less of their Dentists then the best results.

Dr. Woolworth, of New Haven. A Dentist should labor to teach his patient to appreciate and judge of good operating. We have appreciative people to deal with. It is a good test of a Dentist whether he is willing to have his brethren pass judgment on his work or not.

Dr. Atkinson. We are a young body; and it would not be fair to suppose that a majority are up to their best ideal. Educating the people is really the best thing; we can not press that point too strongly; it reacts upon us by keeping us up to the highest notch in our profession. Men who have been A No. 1, but stood still, are drifting down the current; every one who stops advancing grows old fogy, when every man accepts his profession as his divine commission, we shall have a profession which will stand in the van as doers of service and be recognized as such.

Dr. Hurd. It does very well to talk in a general way about equivalents; but what we want is to come to a practical point and say what is an equivalent. It will not answer to count up the whole future value of a saved tooth for the rest of a life time; for then you will certainly be at fault. The salvation of a single tooth may prolong a man's life for years, but will it answer to charge for the saving of that tooth, the price at which your patient may value his life. There is a proper standard of value to be determined by our necessities and varied by the ability of our patients. The rich may command our best services; for such we are entitled to an ample reward. Exorbitant charges are as damnable as pitiful charges. He once filled teeth for a dollar, before he knew what his services were worth; now he had learned to appreciate his work and he charged accordingly. The proper way is to use reason in determining the matter: all can not charge alike, but according to the sphere of their labor.

Dr. Marvin. No subject is so hard to settle as that of Dental charges, because there is no fixed standard of value. People value the services of a Dentist very differently. Some would rather have "a new set" than pay for having their own teeth preserved. This class will not estimate rightly; others will estimate in a matter of fact way. Some Dentists arrange their prices according to the amount of their work: Surgeons do the same; one man charging $10 and another $250 for similar operations. A man must feel he has done a good operation and done it to the best of his ability. Every one knows when he has done this—he can have no better guide than his own consciousness. No class of men work harder than the good Dentist, and such an one is entitled to all he can get: always allowing his humanity to come in and not be over exacting.

Dr. W. H. Allen, said his feelings were entirely with the last speaker; we are not apt to work at losing rates; the operator should feel he had done some good in order to justify him in making any charge. He stated the case of a young man who having had very poor teaching, practiced for a time and became so disgusted with his own performances that he gave up, saying "if this is Dentistry its a humbug," he came to the speaker and told this, and after being better prepared, went to work again knowing what he was about. This young man had a proper appreciation of what he had done. Fees necessarily vary with the sphere in which one practices—the man who has among his patients the best people in the community should charge accordingly. Every one has a right to increase his charges, but his patients should be fore-warned of it. For himself he always liked to be conservative—and found it was best to have a perfect understanding with his patient.

Dr. Hurd. We all have among our patients liberal fellows who are willing to pay well. Let us suppose a case: I operate for Mr. Johnson and charge him good prices, which are cheerfully paid; but Mr. J. may have a poor sister, is it for my interest to charge her the same prices I would Mr. J?

Dr. W. H. Allen. In such a case I would consider whether I wanted to give away $50 worth of work, just as I would in any other case of charity.

Dr. Marvin. In such a case I look to Mr. Johnson to pay his sister's bill.

Dr. Mills, said he had always been anxious to do the best for his patients that lay in his power, he worked on principle, and had got so as to fill teeth without counting the money beforehand. We should all have a high standard; and as our abilities increase our charges may increase, but we must all "creep before we can walk."

Dr. W. H. Allen, called on Dr. Metcalf, President of the New Haven Society. The Dr. said he was happy to hear the views of New York Dentists on the subject of Dental charges, in their city they had entered on a new era in regard to prices; it was hardly possible to fix a scale—though they had essayed to do so. He spoke highly of the advantages derivable from Dental societies, and of the New Haven Society in particular.

Dr. Atkinson said in conclusion. When our Dental School is established there will be opportunities for young men such as never before have been presented.

The company then adjourned to the supper table, where an elegant and delicious repast was in waiting, to which ample justice was done. "Health and prosperity to DR. SMITH," the wish of every one present, was pledged in the sparkling juice of the clear and cool Catawba.

After admiring, where there was no room to criticise, some of the Dr.'s beautiful operations, we were piloted by him through New Haven's classic shades to our point of departure.

With hearty leave-takings, the Society returned to New York to cherish the memory of this, as one of its pleasantest meetings.

